# AKT1 and AKT2 isoforms play distinct roles during breast cancer progression through the regulation of specific downstream proteins

**DOI:** 10.1038/srep44244

**Published:** 2017-03-13

**Authors:** Marina Riggio, María C. Perrone, María L. Polo, María J. Rodriguez, María May, Martín Abba, Claudia Lanari, Virginia Novaro

**Affiliations:** 1 Instituto de Biología y Medicina Experimental (IBYME), Vuelta de Obligado 2490 Buenos Aires (1428), Argentina; 2 Centro de Investigaciones Inmunológicas Básicas y Aplicadas. Fac. Ciencias Médicas – Universidad Nacional La Plata (1900), Argentina

## Abstract

The purpose of this study was to elucidate the mechanisms associated with the specific effects of AKT1 and AKT2 isoforms in breast cancer progression. We modulated the abundance of specific AKT isoforms in IBH-6 and T47D human breast cancer cell lines and showed that AKT1 promoted cell proliferation, through S6 and cyclin D1 upregulation, but it inhibited cell migration and invasion through β1-integrin and focal adhesion kinase (FAK) downregulation. In contrast, AKT2 promoted cell migration and invasion through F-actin and vimentin induction. Thus, while overexpression of AKT1 promoted local tumor growth, downregulation of AKT1 or overexpression of AKT2 promoted peritumoral invasion and lung metastasis. Furthermore, we evaluated The Cancer Genome Atlas (TCGA) dataset for invasive breast carcinomas and found that increased AKT2 but not AKT1 mRNA levels correlated with a worse clinical outcome. We conclude that AKT isoforms play specific roles in different steps of breast cancer progression, with AKT1 involved in the local tumor growth and AKT2 involved in the distant tumor dissemination, having AKT2 a poorer prognostic value and consequently being a worthwhile target for therapy.

Breast cancer is the most common cancer in women and the second cause of death around the world^1^. Despite the improvement in diagnoses and the advances in tumor therapy, cancer deaths are due to tumor dissemination^2^. Perturbations in normal cell-cell or cell-extracellular matrix interactions lead to a disruption in basement membrane integrity and surrounding tissue invasion, a previous step in metastatic progression^3^.

PI3K/AKT/mTOR is the most commonly deregulated pathway in the majority of solid tumors including breast carcinomas^4^,^5^. It has been demonstrated that the overactivation of the pathway contributes to tumorigenesis and tumor progression in the mammary gland through growth factor independent cell proliferation^6^, cell invasion^7^, endocrine receptor deregulation^8^,^9^ and resistance to therapy^10^. It has been demonstrated that PIK3CA, AKT and PTEN present increased mutation rates leading to pathway deregulation^11^. However, the mechanism and downstream signals by which PI3K and AKT regulate each step of tumor development and cancer progression are not completely understood.

In experimental models, PI3KCA mutations have been shown to activate downstream kinase AKT and induce oncogenic transformation^12^,^13^ hence, these lesions render tumors highly sensitive to PI3K/AKT/mTOR-directed therapeutic inhibition. Despite the huge amount of clinical trials targeting the pathway in cancer, the only approved drug is Everolimus, an mTOR inhibitor, in combination with Exemestane for advanced hormone receptor positive (HR+)/HER2-negative (HER2−) breast cancer that progressed on endocrine therapy^14^,^15^. However, the relationship between pathway activation or PIK3CA mutation and clinical outcome in breast cancer is still controversial^16^,^17^,^18^,^19^.

There are three AKT isoforms; AKT1, AKT2 and AKT3, that share high sequence and structural homology but exhibit specific and non-redundant functions in various tumor types^6^,^20^,^21^,^22^. In the breast, the role of AKT3 has been less studied so far, but it seems to have a more preponderant role in triple negative tumors^23^. Regarding AKT1 and AKT2 studies, several transgenic mouse models with mammary carcinomas have been developed, and they showed diverse results. In an ErbB2-induced model, Hutchinson *et al*.^24^ showed that activation of AKT1 accelerates tumorigenesis but suppresses tumor invasion, whereas Ju *et al*.^25^ reported a proliferative and pro-migratory role for AKT1. In MMTV-ErbB2/neu and MMTV-PyMTV models, Maroulakou *et al*.^26^ reported that AKT1 ablation delays tumor formation, but has no effect on metastasis, whereas AKT2 ablation enhances mammary tumor growth. Contrarily, in the MTB-IGF-IR model Watson *et al*.^27^ reported that AKT1 or AKT2 ablation delays mammary tumor onset and suppresses tumor growth.

In mouse and human breast cancer cells our group described that the overactivation of AKT1 leads to ductal-like tumor growth^9^ and hormone-independent activation of endocrine receptors^8^,^28^. Other studies showed that while AKT1 inhibits cell migration by ERK regulation^29^ or by the degradation of NFAT^30^, AKT2 promotes cell invasion through β1-integrin^31^ and palladin upregulation^32^. These and other studies^33^,^34^ have postulated that inhibition of AKT1 can lead to enhanced tumor cell invasiveness and metastatic disease. However, none of the above mentioned human breast cancer models fully discriminated the specific role for AKT1 and AKT2 on the aggressive phenotype and the disease progression *in vivo*. The present study was designed to analyze the specific role and signaling of AKT1 and AKT2 in regulating the different stages of breast cancer progression in two human ductal breast cancer cell lines growing in culture and in xenografts.

## Results

### AKT1, but not AKT2, promotes cell proliferation

It has been shown that the overactivation of AKT1 leads to increased mammary tumor growth^9^,^20^,^25^,^28^. In this study, estrogen and progesterone receptor positive (ER + /PR + ) IBH-6 and T47D cancer cells were stably modified to upregulate or downregulate specifically AKT1 or AKT2 isoforms and search for their specific effects on cell proliferation, adhesion and invasion.

**Figure 1:**
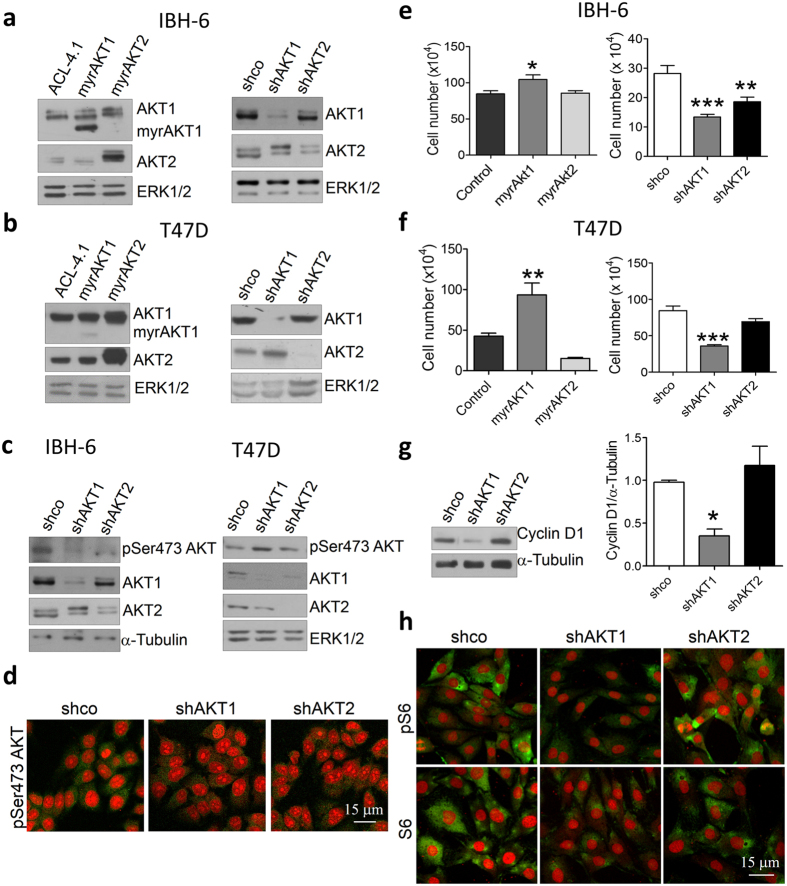
AKT1 promotes cell proliferation in IBH-6 and T47D cell lines. (**a** and **b**) WB showing AKT1 or AKT2 upregulation (left) and downregulation (right) in myrAKT and shAKT cells, respectively. (**c, left**) WB and (**d**) IF in green showing decreased pSer473AKT levels in AKT1 and AKT2 deficient IBH-6 cells. (**c**, right) WB showing decreased pSer473AKT levels in AKT1 and AKT2 deficient T47D cells. The antibody for pSer473AKT recognizes all phosphorylated AKT isoforms in Ser473. (**e** and **f**) Cell proliferation in AKT-overexpressing (left) or -deficient (right) cells. (**g**) WB (left) and quantification (right) for cyclin D1 in IBH-6 AKT-deficient cells. (H) IF in green for pSer240S6 (pS6, upper panel) and total S6 (lower panel) in IBH-6 AKT-deficient cells. Nuclei were counterstained in red with PI. ERK1/2 or α-Tubulin were used as loading controls. *p < 0,05; **p < 0,01; ***p < 0,001 vs. control or shco cells. n = 3 (ANOVA, Dunnet’s post-test). Blots in **a**, **b**, **c** and **g** were cropped. Full-length blots are presented in Supplementary Figure S6.

The overactivation of AKT1 (myrAKT1) or AKT2 (myrAKT2) was generated with a myristoylation sequence, which maintains the constitutively active protein at the cell membrane. Downregulation of AKT1 (shAKT1) or AKT2 (shAKT2) was generated with shRNA constructions. The respective modulation of specific isoforms was corroborated by the evaluation of protein expression (Fig. 1a and b and Supplementary Fig. S1a, b and c) and phosphorylation (Fig. 1c and d and Supplementary Fig. 1d). We could not distinguish isoform-specific regulation in the phosphorylation of pSer473AKT (Fig. 1c and d) and pThr308AKT (Supplementary Fig. 1d) what would indicate differential activation, because these antibodies do not discriminate between the AKT isoforms.

We then analyzed the effect of AKT modulation on cell proliferation. As expected, in both cell lines, AKT1 overexpression increased, whereas its downregulation decreased cell proliferation. However, the effect of AKT2 on cell growth was less clear (Fig. 1e and f). Statistically significant downregulation on cell proliferation was only observed in IBH-6 shAKT2 cells (Fig. 1e, right). Furthermore, the inhibition of cell proliferation in IBH-6 shAKT1 cells was accompanied by cyclin D1 downregulation (Fig. 1g) and by a decrease in S6 expression and phosphorylation (Fig. 1h). However, AKT2 had no effect on cyclin D1 or S6 regulation. Similar results were observed in T47D cells (Supplementary Fig. 1e and f). These results indicate that in IBH-6 and T47D cells, AKT1, but not AKT2, controls cell proliferation through S6 and cyclin D1 regulation.

### AKT1 and AKT2 play different roles in cell migration and invasion

We analyzed the effect of specific AKT downregulation on cell aggressiveness. IBH-6 epithelial cells display a spindle-shaped morphology^35^. In monolayer culture (two dimension, 2D), IBH-6 shAKT1 and IBH-6 shAKT2 cells preserved size and morphology compared to IBH-6 shco cells (Supplementary Fig. S2a). T47D shAKT1 and T47D shAKT2 also preserved size and morphology in 2D (not shown).

**Figure 2:**
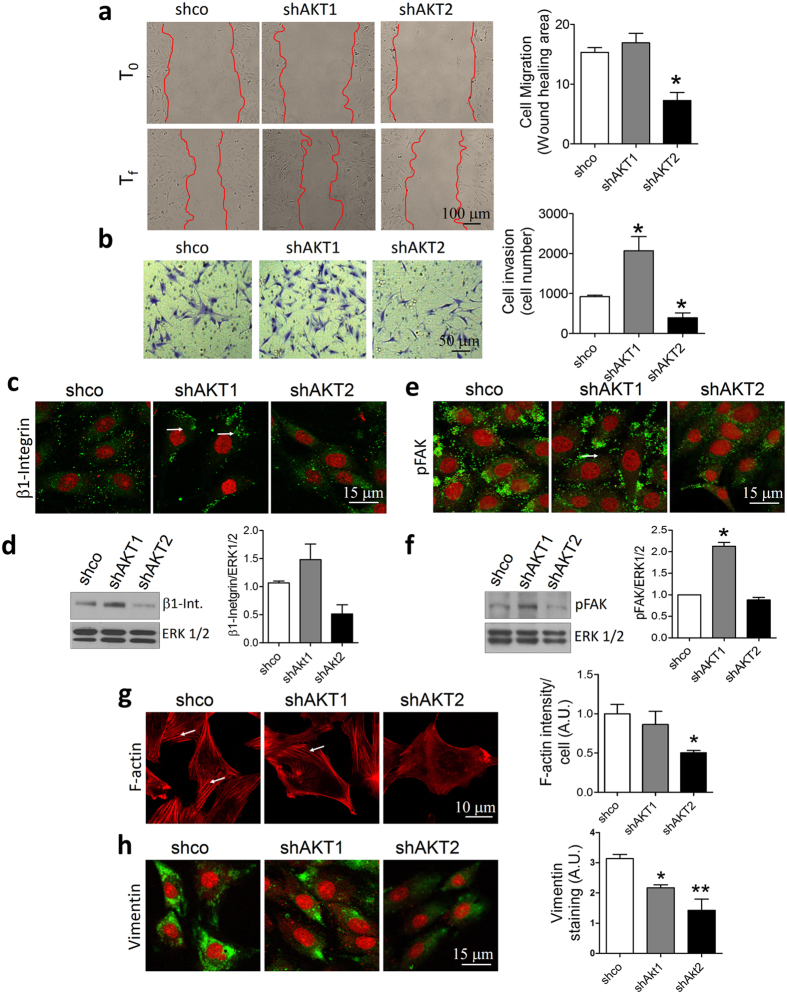
AKT1 and AKT2 play different roles in cell migration and invasion in IBH-6 cells. (**a**) Wound healing assay. Representative pictures at T0 and Tf (21 hours after) of one experiment (left). Bar graphs represent the quantification of the migration area (right). (**b**) Transwell invasion assay (left). Bar graphs indicate the average of cells that invaded the Matrigel and attached on the other side of the insert after 26 hours (right). (**c** and **e**) IF for β1-integrin and pTyr861FAK (pFAK). (**d** and **f**) Representative WB (left) and quantification (right) of β1-integrin and pFAK. (**g**) IF for phalloidin to stain in red F-actin fibers (left) and quantification of F-actin intensity (right). (**h**) IF for vimentin (left) and quantification of vimentin staining (right). Nuclei were counterstained in red with PI (except in **g**). White arrows show punctuated localization (**c** and **e**) and actin fibers (**g**).*p < 0,05; **p < 0,01. n = 3. Blots in **d** and **f** were cropped. Full-length blots are presented in Supplementary Figure S7.

On a three-dimensional (3D) basement membrane Matrigel, IBH-6 shco cells aggregated on top, with some cells invading the Matrigel (Supplementary Fig. S2b). Downregulation of AKT1 increased the invasive phenotype, while downregulation of AKT2 suppressed this phenotype, and the cells remained as aggregates (Supplementary Fig. S2b). Consistently, in a wound-healing assay shAKT1 cells had similar migration capacity than IBH-6 shco cells, whereas shAKT2 cells decreased it (Fig. 2a). Furthermore, shAKT1 increased and shAKT2 reduced the ability to invade the Matrigel (Fig. 2b). The differential effect of AKT1 and AKT2 on the invasive phenotype was less evident in T47D cells (not shown).

We then analyzed the effect of dual isoform silencing (shAKT1/2). For this, IBH-6 cells were co-infected to simultaneously downregulate AKT1 and AKT2 (Supplementary Fig. S3a). ShAKT1/2 cells decreased proliferation (Supplementary Fig. S3b), similar to shAKT1 cells. Moreover, the effect of shAKT1 prevailed over the effect of shAKT2 and the resultant phenotype was an increase in the migration (Supplementary Fig. S3c) and invasive (Supplementary Fig. S3d) capacities of IBH-6 shAKT1/2 cells. These results demonstrate that AKT1 inhibits cell migration and invasion *per se*, and its effect is prevalent over AKT2.

### AKT1 and AKT2 opposite regulation of cellular adhesion and invasion proteins

Integrins belong to a family of cell membrane receptors involved in cellular-extracellular matrix interactions through protein kinases effectors, and in regulating the activity and localization of intracellular and extracellular proteins to favor cellular dissemination^36^,^37^.

In a previous study, we demonstrated that the overactivation of AKT1 downregulated focal adhesion kinase (FAK) expression, leading to a decrease in IBH-6 cell adhesion^9^. In this study, we analyzed β1-integrin and FAK levels after downregulation of AKT1 or AKT2. β1-integrin increased in shAKT1 cells (Fig. 2c and d). Consistently, phosphorylation of FAK (pFAK) increased in shAKT1 cells and did not change in shAKT2 cells (Fig. 2e and f). MMP9 was also increased in shAKT1 cells (not shown). Furthermore, in shAKT1 cells, β1-integrin and pFAK were particularly increased near membrane edges in a punctuated localization (Fig. 2c and e).

We also analyzed the cytoskeleton components F-actin and vimentin, commonly deregulated during cell migration. While IBH-6 shAKT1 cells expressed similar levels of F-actin as shco cells, with clearly defined actin fibers, IBH-6 shAKT2 cells exhibited a weaker pattern of expression, with non-defined filaments (Fig. 2g). Moreover, while IBH-6 shco cells expressed high levels of vimentin, shAKT1 diminished levels of this protein with a polarized pattern (Fig. 2h). ShAKT2 cells also diminished the levels of vimentin, although with a non-polarized pattern, consistent with their decreased ability to invade the Matrigel (Fig. 2h). Even though, neither overexpression of AKT1 nor AKT2 changed cell morphology in 2D (Supplementary Fig. S4a), myrAKT1 cells decreased levels and polarization of pFAK (Supplementary Fig. S4b), whereas myrAKT2 cells increased F-actin polymerization (Supplementary Fig. S4c).Wild type (WT) cells presented a similar behavior than ACL-4.1 or pCDNA3 control transfected cells (not shown). Altogether, these results suggest that cell invasion is regulated differently by AKT1 and AKT2 through their downstream effectors. That is, whereas cellular adhesion proteins β1-integrin and FAK are preferentially inhibited by AKT1, the cytoskeleton components F-actin and vimentin are preferentially induced by AKT2.

### AKT1 and AKT2 generate different breast cancer phenotypes in xenografts

**Figure 3:**
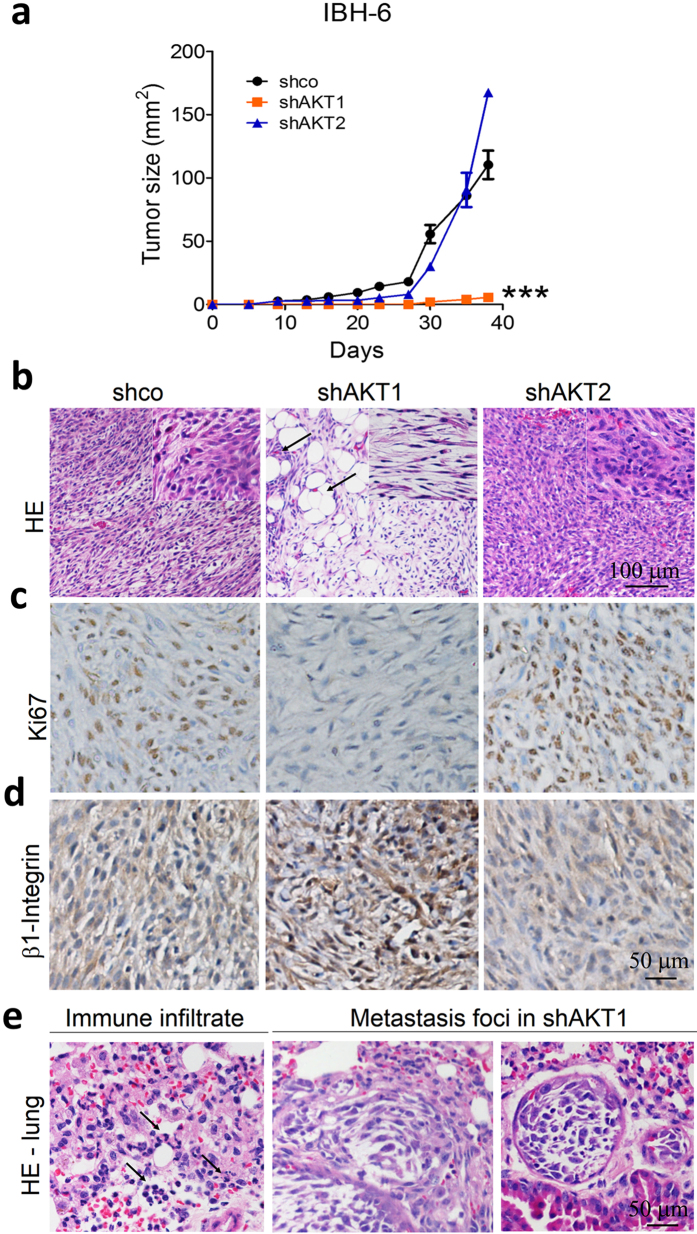
AKT-deficient IBH-6 cells growing as xenografts reproduced in culture differences between AKT1 and AKT2 isoforms. (**a**) Growth curves of IBH-6 shco and AKT-deficient cells in NSG mice. (**b**) HE in tumor samples, arrows indicate tumor invasion of adjacent adipose tissue. IBH-6 shco tumors were poorly differentiated, similar to IBH-6 WT carcinomas, with two cell populations clearly defined: one epithelial-like and other spindle-shaped (inset). (**c**) Ki67 and (**d**) β1-integrin staining. (**e**) HE in lungs from IBH-6 shAKT1 tumor-bearing animals, showing polymorphonuclear cell infiltration (left, indicated by the arrows) and two metastasis foci (right) in 6 out of 12 animals. ***p < 0,001. n = 4 in each experimental group for tumor growth curves. Experiments were performed by duplicate.

In a previous work, we have shown that IBH-6 myrAKT1 cells injected into immunosuppressed animals generated tumors that grow faster than IBH6 ACL-4.1 cells^9^. Here, we show that IBH-6 shAKT1 cells originated smaller tumors with a lower growth rate compared to IBH-6 shco cells (Fig. 3a). On the contrary, IBH-6 shAKT2 cells did not change tumor latency or growth rate. It is worthwhile to mention that IBH-6 shco cells generated tumors that grow similarly to IBH-6 WT cells^38^ and that all IBH-6-derived cell lines grow in the absence of exogenous hormone supply. Because IBH-6 shco and shAKT2 tumors grew so fast, when they reached the maximum permitted size according to NIH guidelines and^39^, the animals were sacrificed, and tumors, lungs and liver were removed to analyze the histology and the presence of metastasis.

The tumor histology showed relevant differences depending on the specific AKT isoform downregulated (Fig. 3b). IBH-6 shAKT1 tumors were enriched in the spindle fibroblastic-like population, with almost no epithelial cells (Fig. 3b, inset). Furthermore, despite the reduced growth rate, shAKT1 tumors displayed evident signs of invasion of the adjacent adipose tissue (Fig. 3b, arrows). Contrarily, IBH-6 shAKT2 tumors displayed a less spindle-shaped morphology, with no signs of invasion (Fig. 3b). We analyzed by IHC the expression of AKT1 and AKT2 in shco and AKT-deficient tumors (Supplementary Fig. S5a and b) to confirm the specific AKT dowregulation. ShAKT1 tumors displayed fewer Ki67-positive cells (Fig. 3c) consisting with a reduced proliferation rate (Fig. 3a). Finally, the β1-integrin level in the tumors reproduced the results obtained in cell cultures; that is, increased levels in shAKT1 and decreased levels in shAKT2 tumors, compared to shco tumors (Fig. 3d).

At the end of the experiment, 40 days after cell inoculation, no foci of metastasis were found in any of the experimental groups (not shown). Nevertheless, the lungs from shAKT1 tumor-bearing animals showed signs of inflammation, such as polymorphonuclear cell infiltration (Fig. 3e, left), suggesting a favorable pre-metastatic niche for the circulating cells. Long-term experiments (60 days after cell inoculation) with IBH-6 shAKT1 cells generated lung metastasis (Fig. 3e, right). Long-term experiments were not possible in shco or shAKT2 tumor-bearing animals because of animal welfare concerns.

**Figure 4:**
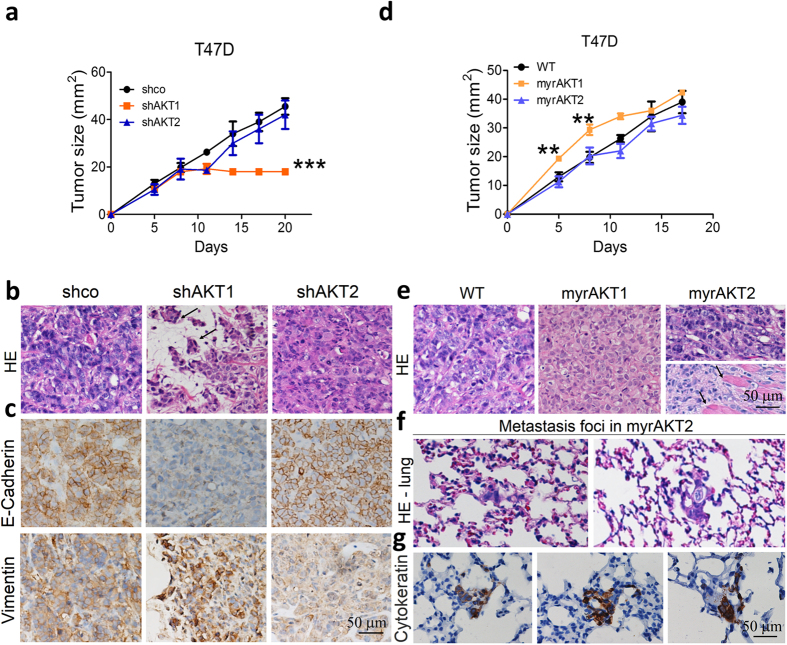
AKT-expressing T47D xenografts confirm the differential effect of AKT1 and AKT2 on tumor growth and invasion. Growth curves of (**a**) T47D shco and AKT-deficient cells or (**d**) T47D WT and AKT-overexpressing cells in NSG mice. (**b** and **e**) HE in tumor samples, arrows indicate tumor invasion of adjacent adipose or muscle tissue. (**c**) E-cadherin and vimentin staining in T47D shco and AKT-deficient tumors. (**f**) HE in the lungs of T47D myrAKT2 tumor-bearing animals showing metastasis foci in 8 out of 12 animals. (**g**) IHQ for pan-cytokeratin stains epithelial metastatic cells in the lungs. **p < 0,01; ***p < 0,001. n = 3 in each experimental group for tumor growth curves. Experiments were performed by duplicate.

Similarly, in T47D xenografts AKT1 silencing reduced tumor growth, whereas AKT2 silencing did not change the growth rate compared to control tumors (Fig. 4a). Even though T47D shAKT1 tumors were smaller in size, were the only ones with evident signs of invasion, with groups of cells invading the adjacent adipose tissue (Fig. 4b, arrows). AKT isoform-specific downregulation was confirmed by IHC (Supplementary Fig. S5c and d). T47D shAKT1 tumors expressed low levels of E-cadherin and high levels of vimentin, which could be a prelude of a stemness phenotype, whereas shAKT2 tumors showed the opposite pattern, high E-cadherin and low vimentin (Fig. 4c). No metastasis foci were observed at the time of sacrifice in any animal with the silenced isoforms (not shown).

To confirm the results of the differential invasive function of AKT isoforms, we overexpressed the specific isoforms. T47D myrAKT1 xenografts displayed lower tumor latency and increased growth (Fig. 4d), as previously observed in IBH-6 myrAKT1 xenografts^9^. However, after 15 days, the growth rate of T47D myrAKT1 tumors was comparable to that of T47D WT tumors (Fig. 4d). This slowdown in the growth rate of T47D myrAKT1 cells could be because these cells are still dependent on the estradiol supply for growth. T47D myrAKT2 xenografts displayed similar growth rate as WT xenografts (Fig. 4d), but in fact, they displayed a histology resembling lobular tumors with invasion of adjacent muscle tissue (Fig. 4e, arrows). Furthermore, long-term experiments (60 days after cell inoculation) showed metastasis foci in the lungs of T47D myrAKT2 tumor-bearing animals (Fig. 4f) positive for the epithelial marker cytoqueratin (Fig. 4g). It is worthwhile to mention that T47D WT is not a metastatic cell line.

### AKT1 and AKT2 tumor levels differentially correlate with the survival in invasive breast carcinomas

**Figure 5:**
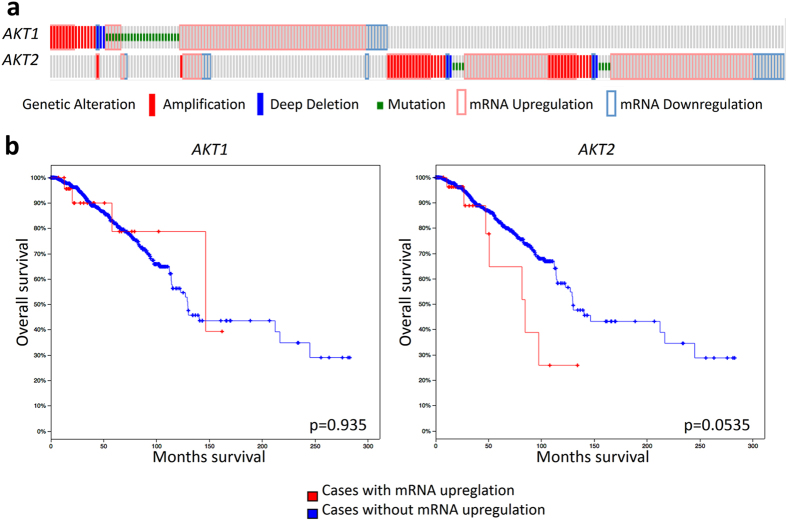
AKT2 has worse prognosis value than AKT1 in invasive breast carcinomas. (**a**) AKT1 (alteration rate 11%) or AKT2 (alteration rate 10%) genetic alterations in 1105 breast invasive carcinoma samples from TCGA. For AKT1 and for AKT2 the predominant alteration in patient samples was upregulation of mRNA. (**b**) Kaplan-Meier curves in the reference population showing that mRNA upregulation of AKT2 was associated with worse overall survival (p = 0.0535) than mRNA upregulation of AKT1 (p = 0.935).

Finally, to assess whether the AKT1 and AKT2 tumor levels are related to breast cancer progression in patients, we evaluated 1105 samples of invasive breast carcinoma available from The Cancer Genome Atlas (TCGA) website^40^,^41^ consisting of data sets with DNA amplifications, mutations, deletions and mRNA up- and downregulations (Fig. 5a). Considering only mRNA expression data profiles for AKT1 and AKT2, we found that AKT2 but not AKT1 was associated with lower overall survival (Fig. 5b).

Altogether, our findings from experimental models and the TCGA meta-analysis support the idea that tumor progression in breast cancer differentially involves AKT1 and AKT2 isoforms. In conclusion, AKT1 and AKT2 level might be used as biomarkers of breast cancer progression early in the diagnosis process to discriminate which subset of patients could progress sooner and consequently have potentially worse clinical outcomes. We provide experimental and clinical evidences that it is mainly AKT2 overexpression that is related to disease progression and has a worse prognostic value in breast cancer.

## Discussion

**Figure 6:**
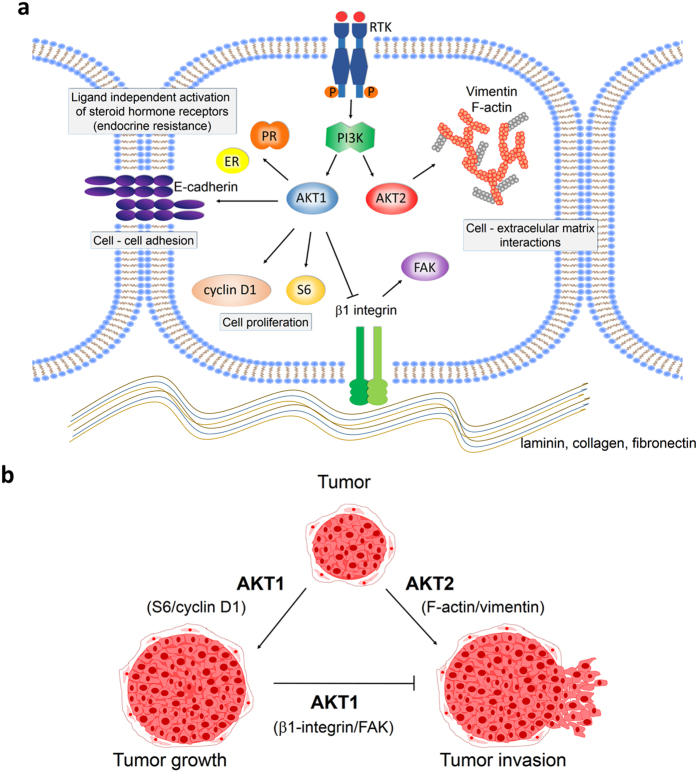
Proposed mechanisms for AKT1 and AKT2 downstream regulation during breast cancer progression. (**a**) Specific signaling of AKT1 and AKT2 and particular downstream protein regulation. (**b**) Representative scheme for AKT1 and AKT2 specific functions on tumor growth and tumor invasion.

Our results show that AKT1 and AKT2 isoforms regulate breast cancer cells differently. Here we describe the mechanisms by which AKT1 and AKT2 play specific roles in tumor growth and dissemination in IBH-6 and T47D human cell lines. We found that AKT1 is relevant for cell proliferation and survival through S6 and cyclin D1 upregulation. Both AKT1 and AKT2 are involved in cell migration and invasion, although each isoform differentially regulates β1-integrin, FAK, E-cadherin, F-actin and vimentin expression (Fig. 6a) and consequently have opposite effects on the aggressive phenotype (Fig. 6b). AKT1- and AKT2-specific cell functions could be appreciated also *in vivo* transplantation, and we describe invasion of adjacent adipose and muscle tissues as well as lung metastasis derived from shAKT1 and myrAKT2 xenografts.

Despite the increasing amount of studies regarding the role of the AKT isoforms in cancer, their specific roles are still not clearly elucidated, being the results cell type and context dependent. This highlights the relevance of understanding the specificity of each AKT isoform during cancer progression to better define their prognostic value. Most experimental studies to date have made use of hyperactive variants of AKT such as a membrane-targeted mutants, or knockdown strategies such as shRNA *in vitro*. With this approach, the distinct roles of AKT1 as a growth inducer and AKT2 as a growth and invasion repressor were well defined in ovarian cancer cells, both within the tumor and the microenvironment^22^. In the breast, most data in transgenic mouse models agree that AKT1 is critical for breast cancer induction whereas AKT2 is more involved in the metastatic dissemination^20^,^26^.

In breast and ovarian cancer cells, AKT2-induced migration and invasion are linked to the upregulation of β1-integrin^31^, however, in prostate cancer cells, both AKT1 and AKT2 reduced cell migration and invasion through downregulating β1-integrin^42^. Here we show in IBH-6 cells that the specific downregulation of AKT1, but not AKT2, increased β1-integrin expression and FAK phosphorylation in punctuated membrane areas compatible with focal adhesion sites. FAK can be considered up and downstream of AKT in the regulation of growth factor and integrin-stimulated cell motility. We have previously shown in the IBH-6 cells that the overactivation of AKT1 decreased, whereas the overexpression of PTEN increased FAK levels^9^. In contrast, in human colon cancer cells, Wang *et al*.^43^ reported that AKT1, but not AKT2, directly binds to and phosphorylates FAK. Moreover, here we showed the involvement of AKT2, but not of AKT1, in the remodeling of the actin cytoskeleton, which is also relevant to cancer cell migration. Altogether, our results confirm that AKT2 has a promigratory role in breast cancer cells.

Several studies have considered E-cadherin as a tumor suppressor gene, suggesting that its decreased expression is a requirement for tumor progression. The overexpression of AKT1 in MCF10A cells grown in 3D Matrigel and injected into the mouse mammary duct, resemble ductal carcinoma *in situ*-like lesions^44^. Similarly, we have previously reported that AKT1 is involved in ductal differentiation in 3D cultures and *in vivo*^9^ and that myrAKT1 breast cancer cells form organized and polar-like structures with high E-cadherin expression. Here we show that AKT-specific-deficient T47D tumors display the inverse regulation of E-cadherin and vimentin. Despite the loss of E-cadherin, AKT1-defficient tumors maintain cohesive invasive cells with higher levels of vimentin. We hypothesize that it is most likely the balance between invasive proteins rather than the protein level in individual cells, which determines the invasive grade of a tumor. Overall, we speculate that AKT1 functions as an invasion suppressor during the early phases of the disease, whereby AKT2 enhances cell invasion and aggressiveness in advanced phases of the disease (Fig. 6b).

Finally, we found, in a univariate analysis of 1105 samples of breast invasive cancer from TCGA, that patients that carry mutations that increase abundance of AKT1 mRNA level have a better outcome than patients with mutations at the level of AKT2, who have shorter overall survival. Consequently, our study proposes that AKT2 constitutes a prognostic marker of poor clinical outcomes in breast cancer. This conclusion is further supported by the cellular mechanisms we described here in two human breast cancer cell lines.

Recent studies that have associated AKT expression with cancer prognosis showed some contradictory results depending on the characteristics of the population evaluated and the technical approach^45^,^46^,^47^. Pereira *et al*.^47^ pointed out that AKT2 is involved in the acquisition of stem cell-like properties, responsible for invasiveness and chemoresistance and worst breast cancer outcome. However, Fohlin *et al*.^46^ demonstrated that AKT2 and pAKT (pSer473AKT) levels were associated with a lower distant recurrence rate. In the same line, Grell *et al*.^45^ showed that AKT2 expression and concurrent presence of pAKT (pThr308AKT and/or pSer473AKT) linked to better outcome in HER2 + metastatic patients treated with Trastuzumab. Moreover, Badve *et al*.^48^ found that nuclear localization of pAKT (pSer473AKT) was associated with long-term better survival in ER + /PR + breast cancer patients. Other studies suggested that AKT1 drives progression in early breast cancer, while AKT2 reverses this effect^49^,^50^,^51^. Furthermore, Plant *et al*.^52^ described a shuffle in AKT1 from the nucleus to the cytoplasm during breast tumor progression. This event could be also related to differential protein activation in tumor tissue as we report here; i.e. proliferation proteins in early stages and ECM remodeling proteins, as β1-integrin, in advanced stages. All these differing evidences advise that a robust assessment of AKT1 and AKT2 in early and late stages of the disease is suggested as new prognostic markers. In prostate cancer patients, AKT expression and phosphorylation were significantly associated to unfavorable outcome, with cytosolic AKT1 expression correlated with a higher risk of postoperative recurrence^53^ and pAKT levels predictive of poor clinical outcome^4^.

Finally, the efforts to dissect the molecular mechanisms of AKT isoform-specific signaling will provide new insights for designing more effective and selective therapeutics for cancer treatment^23^,^54^,^55^,^56^,^57^. In this regard, isoform-specific siRNA, microRNAs, inhibitors and antibodies constitute tools to elucidate the relative contributions of each AKT isoform signal and localization to cancer spreading. Our results in breast cancer cell lines show that even though AKT1/AKT2 dual silencing reduces cell proliferation, it surprisingly enhances cell migration and invasion. Overall, our results suggest that targeting at the PI3K or AKT1/2 level would not be effective in preventing tumor progression, although specifically targeting AKT2-driven signals could be more effective at preventing cancer aggressiveness. The challenge now is to try new target therapies directed to AKT2-activated downstream signaling in combination with drugs that target PI3K and/or mTOR to achieve optimal efficacy in decreasing breast cancer progression. PanAKT inhibitors including MK2206, AZD5363 are currently in clinical trials for advanced solid malignancies such as pancreatic, colorectal, breast, and prostate cancers. Selective inhibitors for the AKT isoforms have been developed and tested preclinically but have not yet reached clinical trials (i.e. CCT128930 with greater selectivity for AKT2 and antitumor activity)^58^. The effect of these or other AKT inhibitors in cell aggressiveness deserves further experimental investigation in the light of current data suggesting that the AKT isoforms have particular roles. Our study suggests that specific disruption of AKT2 may be preferable to panAKT inhibition for the treatment of advanced breast cancer.

## Conclusion

Our findings provide a rationale to guide the use of AKT1 and AKT2 as biomarkers of disease progression and potential targets for new therapies. Their use as prognostic indicators highlights the importance of accurately assessing the expression of PI3K/AKT/mTOR components if this oncogenic signaling is targeted in the clinical setting. Furthermore, the identification of the downstream components of AKT1 and AKT2 signaling involved in each step of the disease will facilitate the implementation of new biomarker-driven therapies to delay or block progression.

Based on the results shown here, our prediction is that PI3K, panAKT or specific AKT1 inhibition may not be recommended, and may even be contraindicated in breast cancer, as it might promote tumor invasiveness and cancer dissemination. Contrarily, knockout of AKT2 or its downstream molecules could be a better option to improve breast cancer treatment outcomes, at least for metastatic disease.

## Materials and Methods

### Cell lines and culture

IBH-6 cell line is derived from a 34 years old pre-menopausal woman^35^, it expresses ER and PR and grows in NSG animals without hormone supply^38^. T47D human cell line requires a previous injection of 0,25 mg of 17β-estradiol to grow in NSG mice^28^.

Both cell lines were maintained in DMEM F12 (Sigma Aldrich St. Louis, MO) 10% fetal bovine serum (FBS, Natocor Córdoba Argentina) medium. For 3D cultures cells were seeded on Matrigel (BD Biosciences San Jose, CA).

### *In vitro* studies

#### Transfections and infections

IBH-6 and T47D cell lines were stably transfected to overexpress AKT1 (pACL4.1-myrAKT1^9^), or AKT2 (pCDNA3-myrAKT2, Addgene #9016). Transfections were performed with Lipofectamine Reagent (Invitrogen) following manufacturer´s instructions. Stable deletion of AKT1 or AKT2 was performed using specific lentiviral shRNA (TRCN0000022937 for AKT1 or TRCN0000055260 for AKT2, Sigma). Shc-002 Non-Target shRNA was used as control vector. For lentiviral preparation specific shRNA was cotransfected with pCMV-dR 8.74 packaging plasmid and pMD2.G plasmid into HEK239T cells. After two days lentiviral particles were collected and cells were transduced in the presence of Polybrene (Sigma). Cells stably transfected were selected with 400 μg/ml Geneticin (G418, Invitrogen) or 5 μg/ml Puromicin (Calbiochem) according to selection gene in each case.

#### Proliferation assay

4 × 10^4^ cells were seeded and maintained during 4 days in DMEM F12 5% SFB medium (for IBH-6) or 5 days in DMEM F12 2% charcoal stripped FBS (chFBS) + 20 nM insulin (for T47D). The medium was changed every other day. At the end of the incubation cells were tripsinized and counted in Neubauer chamber.

#### Wound healing assay

5 × 10^5^ cells were seeded 24 hours prior to the experiment. Wound was made with a tip and incubated for 21 hours in DMEM F12 5% FBS medium. Photographs were taken all along the wound at the beginning (T0) and at the end of the experiment (Tf). Wound healing areas were quantified as T0-Tf using the ImageJ software.

#### Transwell assay

5 × 10^4^ cells were seeded on top of 8 μm transwell inserts (BD Biosciences) previously coated with 1:6 Matrigel: DMEM F12 medium and incubated for 26 hours. Cells were fixed with cold methanol, stained with Cristal Violet 0,1% and the cells that passed through the insert were quantified.

#### Western blots (WB)

Total protein extracts were obtained using RIPA buffer (Sigma). 100 μg of protein from each sample was separated in 10% polyacrylamide electrophoresis gels. Phosphorylated protein’s bands were normalized using total proteins, with the density of bands from the control group set arbitrarily to 1.0 using the ImageJ software.

#### Immunofluorescence (IF)

Cells were fixed with PFA 4%, blocked with PBS 10% FBS and incubated with primary antibodies overnight. After washing with PBS, cells were incubated with FITC or Dylight secondary antibodies (Vector Laboratories Burlingame, CA) and nuclei were counterstained with propidium iodide (PI) or 4′,6-diamino-2-fenilindol (DAPI). Stained cells were analyzed under a Nikon Eclipse E800 Laser Confocal Microscope.

#### PathScan Intracellular Signaling Array Kit

PathScan Intracellular Signaling Array Kit (Chemiluminescent Readout) #7323 (Cell Signaling Technology) was performed following manufacturer´s instructions for IBH-6 and T47D cell lines modified to overexpress or dowregulate AKT isoforms.

### *In vivo* studies

#### Animals

NOD/LtSz-scid/IL-2Rgamma null mice (NSG) from The Jackson Laboratory (Bar Harbor, ME) were bred at IByME Animal Facility and two-month-old virgin females were used for the experiments. Animal care and manipulation were performed in agreement with the International Guidelines and Regulations from the National Institute of Health. IByME’s Ethical Committee approved the experiments and the use of animals for this work CE 023-June 2014.

#### Xenografts and immunohistochemistry (IHQ)

For IBH-6 xenografts, mice were injected subcutaneously with 5 × 10^6^ cells. For T47D xenografts, mice were implanted subcutaneously with silastic pellets containing 17β-estradiol (0.25 mg) and injected subcutaneously with 8 × 10^6^ cells mixed with Matrigel. When tumors were palpable, sizes were measured with caliper Vernier and growth curves were performed plotting tumor size (mm^2^) vs. time.

Tumors, lungs, liver, uterus and ovaries were fixed in 10% formalin, paraffin embedded (FFPE) and sectioned into 5 μm for histochemical analysis. Tumor and tissue histology were evaluated in hematoxylin/eosin (H&E)-stained slides. For IHQ, FFPE tissues were stained for primary antibodies. Primary antiserum was detected after incubation with a biotinylated secondary antibody (Vector Laboratories Inc.) using the Vectastain Elite ABC Kit (Vector Laboratories Inc.) and the diaminobenzidine (DAB) Chromogen and Substrate Buffer (Dako, Agilent Technologies). After IHC, the specimens were counterstained with hematoxylin, dehydrated, and mounted.

#### TCGA analysis

Open data from 1105 breast invasive carcinoma samples from The Cancer Genome Atlas dataset (TCGA)^40^,^41^ and cbioportal platform (http://www.cbioportal.org) were used to link AKT alterations to clinical outcomes. For the survival analysis we considered mRNA expression data profiles by RNA Seq for AKT1 and AKT2 and an arbitrary overexpression level greater than three standard deviations from the mean in the reference population.

#### Antibodies

Total AKT1/2/3 (8312), phosphorylated Ser473AKT1/2/3 (7985), ERK1/2 (94), β1-integrin (8978), cyclin D1 (753) and actin (1616) were purchased from Santa Cruz Biotechnology; total AKT1 (2938), total AKT2 (3063), phosphorylated Ser240/244S6 (2215) and E-cadherin (3195) from Cell Signaling Technology, phosphorylated Tyr861FAK (4804) from Abcam, vimentin (V6630) from Sigma, alpha-tubulin (MS-719) from Neomarkers and cytokeratin Clones AE1/AE3 (M3515) from Dako.

### Statistical analyses

Statistical analyses were performed with the GraphPad Prism™ software 6.0 (GraphPad, Inc. CA). One way ANOVA followed by Tukey or Dunnet´s post-tests was used to compare means of multiple experimental groups. When comparing the means of two different groups, two-sided Student’s *t*-test was applied. Tumor growth curves were studied using regression analysis, and the slopes compared using analysis of variance. In all graphs, the mean ± SEM are shown.

## Additional Information

**How to cite this article**: Riggio, M. *et al*. AKT1 and AKT2 isoforms play distinct roles during breast cancer progression through the regulation of specific downstream proteins. *Sci. Rep.* **7**, 44244; doi: 10.1038/srep44244 (2017).

**Publisher's note:** Springer Nature remains neutral with regard to jurisdictional claims in published maps and institutional affiliations.

## Supplementary Material

Supplementary Material
