# Supplementary material for: AKT1 and AKT2 isoforms play distinct roles during breast cancer progression through the regulation of specific downstream proteins
Source: Sci Rep. 2017 Mar 13;7:44244. doi: 10.1038/srep44244 (PMC5347151; doi:10.1038/srep44244)
Supplement: Supplementary Material [file srep44244-s1.pdf]

AKT1 and AKT2 isoforms play distinct roles during breast cancer progression through the regulation of specific downstream proteins

Marina Riggio[a], María C Perrone[a], María L Polo[a], María J Rodriguez[a], María May[a], Martín Abba[b], Claudia Lanari[a] and Virginia Novaro[a*].

a. Instituto de Biología y Medicina Experimental (IBYME), Vuelta de Obligado 2490 Buenos Aires (1428), Argentina.

b. Centro de Investigaciones Inmunológicas Básicas y Aplicadas. Fac. Ciencias Médicas – Universidad Nacional La Plata (1900), Argentina.

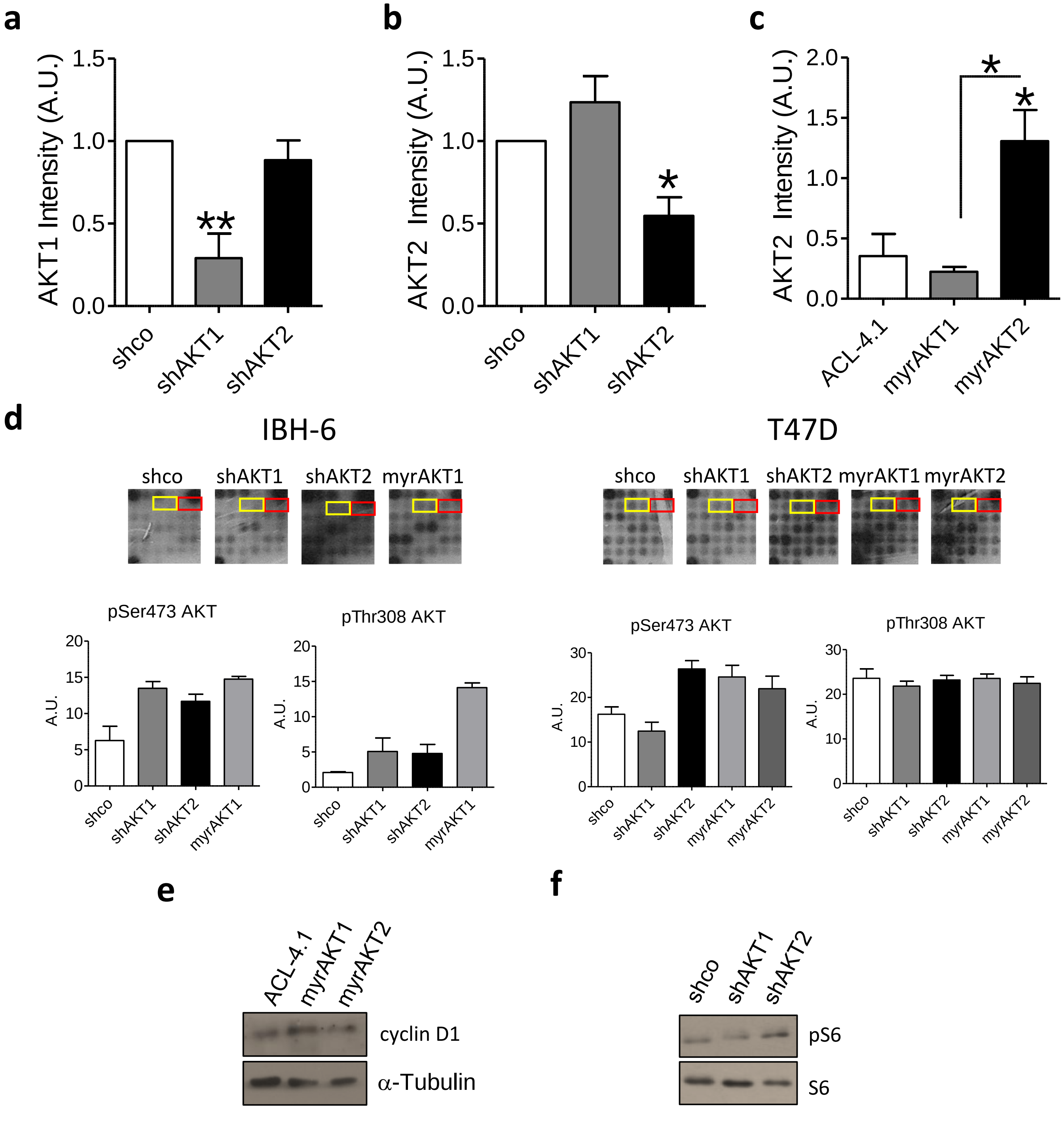

Supplementary Figure S1: AKT modulation in IBH-6 and T47D cell lines. WB quantification showing (**a**) AKT1 intensity and (**b**) AKT2 intensity in AKT-deficient IBH-6 cells. (**c**) AKT2 intensity in AKT-overexpressing IBH-6 cells. AKT1 intensity was not quantified in this cells because the myrAKT1 construction has a different molecular weight. (**d,** upper) PathScan Intracellular Signaling Array Kit for IBH-6 (left) and T47D (right) cells modified to overexpress or downregulate AKT isoforms. Yellow box indicate pThr308 AKT dots and red box indicate pSer473 AKT dots. (**d,** lower) pSer473 AKT (left) and pThr308 AKT (right) quantification in each cell line. (**e**) WB showing cyclin D1 expression in T47D AKT-overexpressing cells. (**f**) WB showing pS6 and S6 expression in T47D AKT-deficient cells.

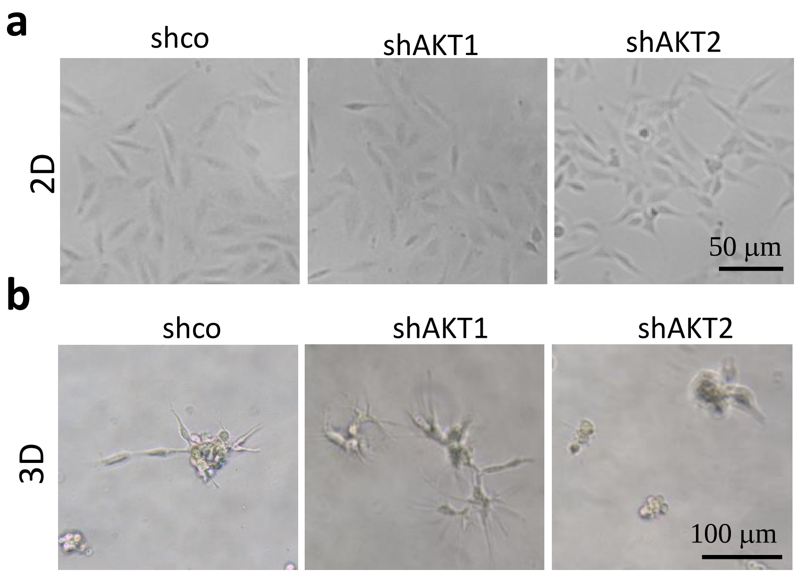

Supplementary Figure S2: AKT1- and AKT2-regulated cell morphology. IBH-6 AKT-deficient cells growing on (**a**) plastic (2D) or (**b**) tridimensional (3D) Matrigel for 48 hours.

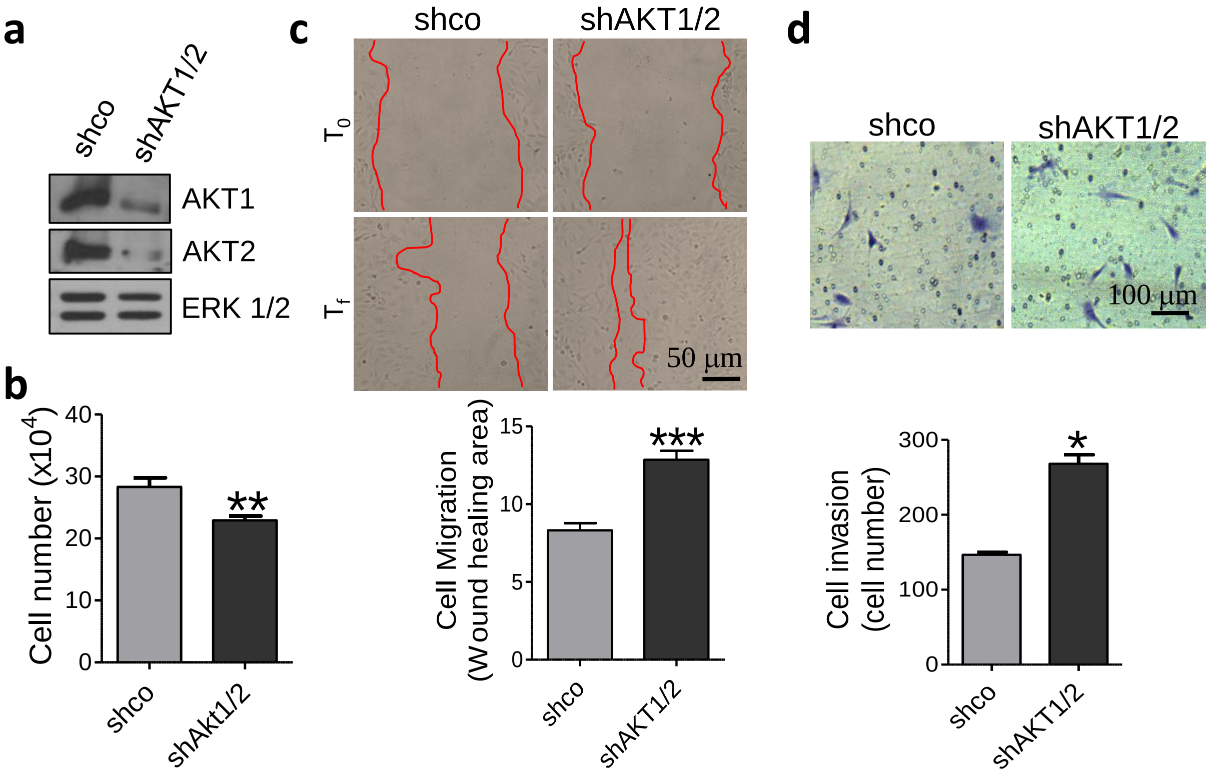

Supplementary Figure S3: AKT1/AKT2 simultaneous silencing enhances cell migration and invasion. (**a**) WB showing simultaneous downregulation of AKT1 and AKT2 isoforms in IBH-6 shAKT1/shAKT2 cells. IBH-6 shAKT1/shAKT2 cells decreased (**b**) proliferation but increased (**c**) migration and invasion (**d**). Representative pictures at T0 and Tf of one migration experiment are shown (c, upper). Representative pictures of one transwell invasion assay in shco and shAKT1/2 cells (d, upper). Bar graphs represent the quantification of the migration area (c, bottom) and the average of cells attached on the other side of the insert (d, bottom). * p<0,05; ** p<0,01; *** p<0,001. n=3.

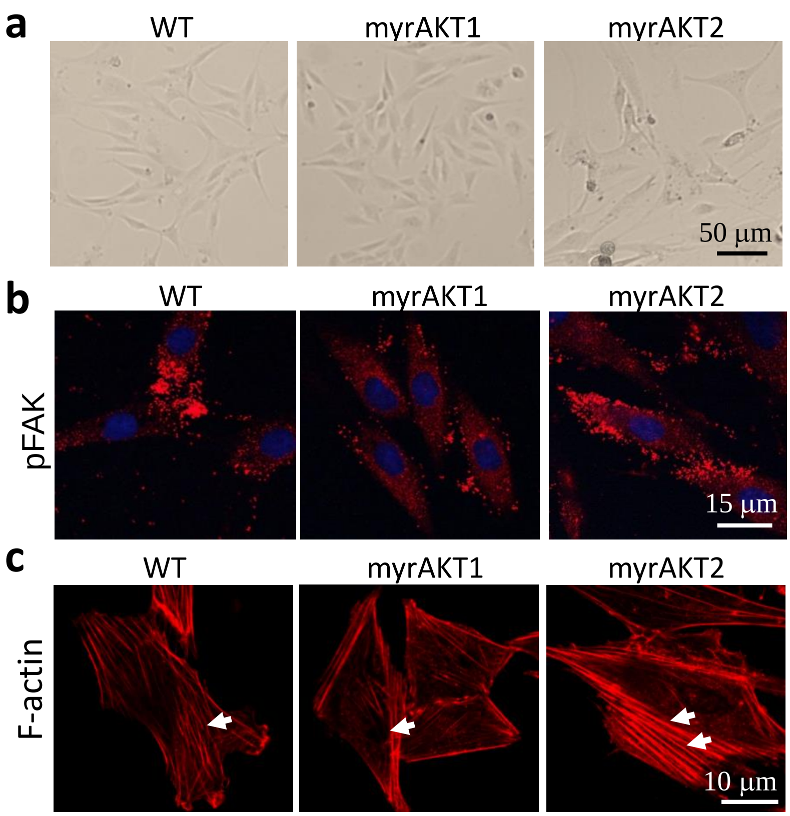

Supplementary Figure S4: AKT1 and AKT2 overexpression modulate adhesion and cytoskeletal proteins. (**a**) Morphology of IBH-6 AKT-overexpressing cells growing on 2D. (**b**) IF in red showing a decreased pTyr861FAK (pFAK) staining in myrAKT1 cells and (**c**) an increase in F-actin fibers polymerization (indicated with white arrows) in myrAKT2 cells stained with phalloidin. In (b) nuclei were counterstained in blue with DAPI.

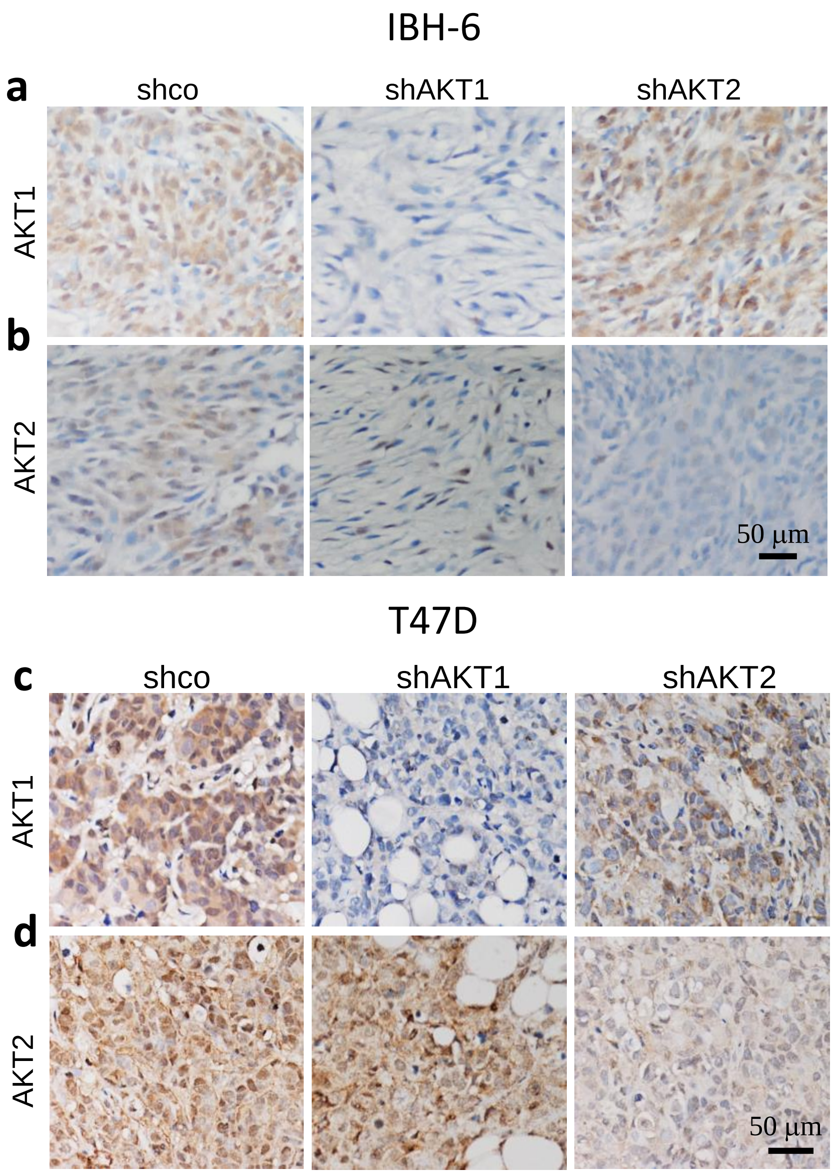

Supplementary Figure S5: AKT modulation in IBH-6 and T47D xenografts. IHC for (**a** and **c**) AKT1 and (**b** and **d**) AKT2 to confirm the specific silencing of each isoform in IBH-6 and T47D xenografts, respectively.

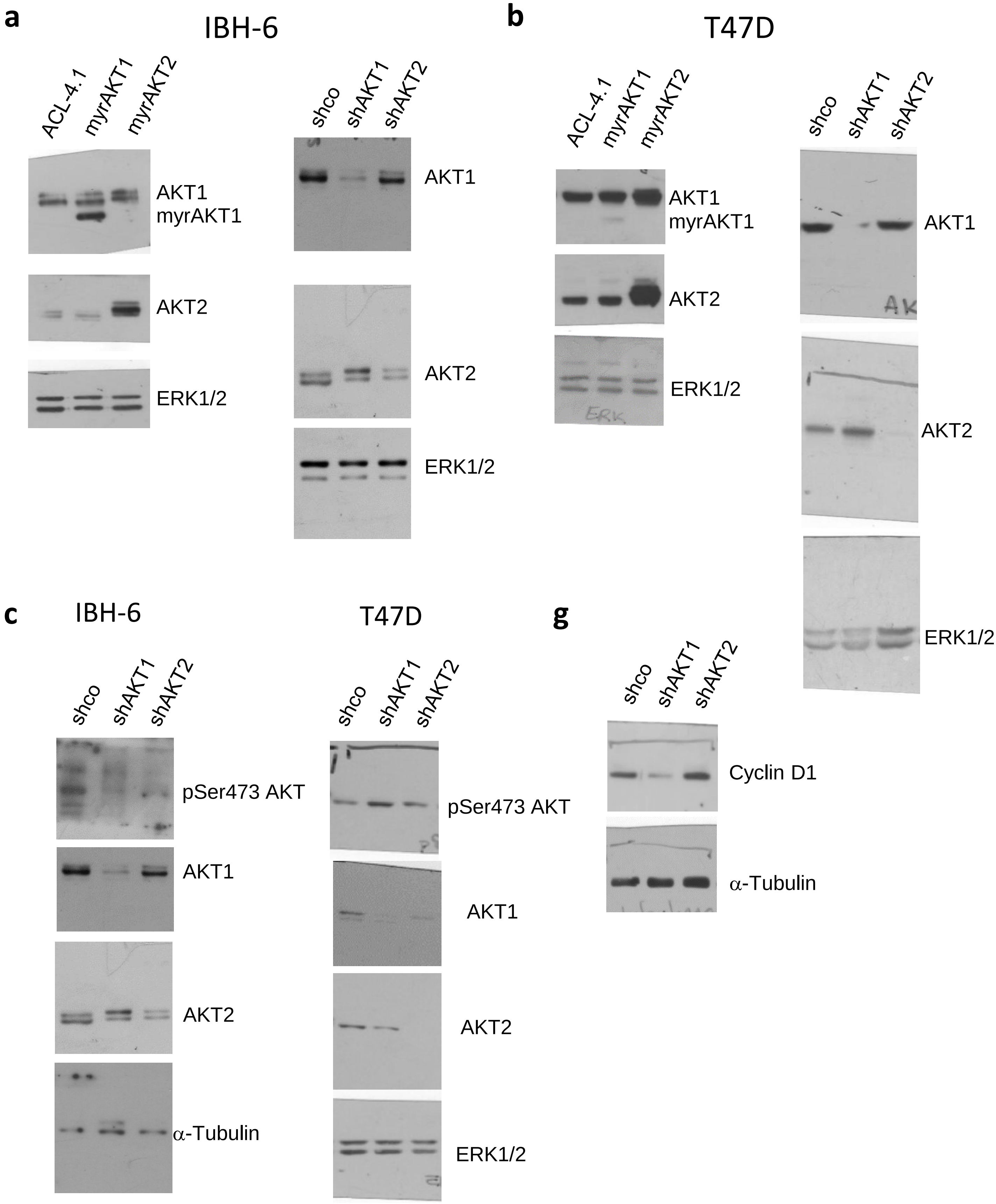

Supplementary Figure S6: Full-length gels from Figue 1.

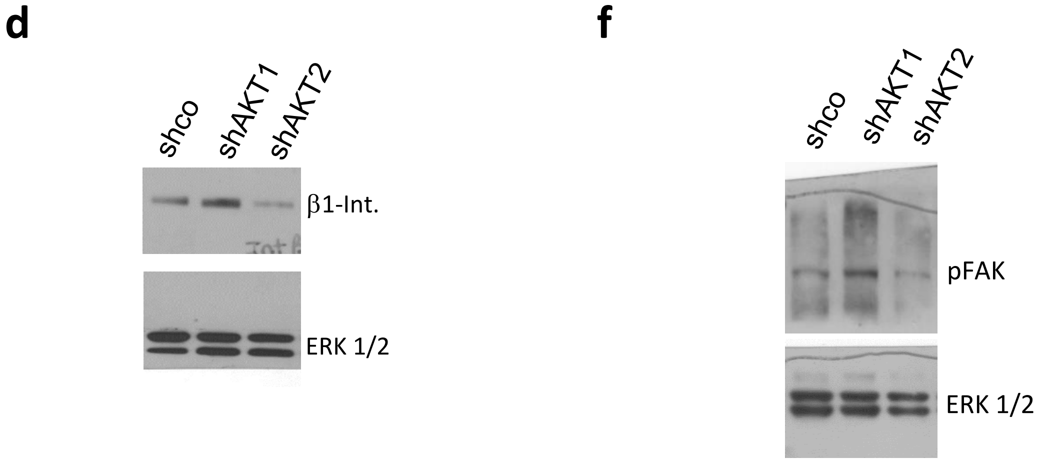

Supplementary Figure S7: Full-length gels from Figue 2.
